# Novel Therapeutic Targets and Approaches, Genetic Markers and Signaling Pathways in Neoplasms—An Overview

**DOI:** 10.3390/cells15191805

**Published:** 2026-10-02

**Authors:** Daria Chudakova

**Affiliations:** Engelhardt Institute of Molecular Biology, Russian Academy of Sciences, 119991 Moscow, Russia; daria.chudakova@eimb.ru

Cancer remains one of the major global health problems. According to the World Health Organization, about 20% of the world population is expected to develop cancer at some point in life. Alarmingly, despite all the significant advances in translational oncology, several key problems persist. Some cancers are still incurable, and for others, the main mechanisms of pathogenesis and therapy resistance have yet to be unraveled. In addition, cancer therapy may cause multiple serious side effects that are poorly tolerated by patients. The high cost of many anticancer therapies poses a significant burden on the healthcare system, patients, and their families. Diagnostic tools for certain cancers, especially rare ones, are limited, leading to late diagnosis and a missed opportunity for early and potentially patient-tailored treatment. Thus, novel, transformative therapeutics and diagnostic strategies are urgently needed, based on comprehensive studies into the genetic, molecular, and cellular characteristics of cancer.

This Special Issue (SI) provides an overview of the emerging intervention targets and approaches, genetic markers and signaling pathways involved in cancerogenesis, as well as the relevant animal models, all of which may lead to novel curative and diagnostic options.

The papers in this SI address some of the key trends in modern translational oncology: personalized therapies that take into account the interplay between the patient’s genetic profile and the impact of environmental exposures (environmental carcinogenesis and toxicogenomics) [1]; the establishment and characterization of relevant animal models to study cancer [2]; the discovery and further development of potential anticancer drug candidates [3,4]; the identification of novel therapeutic targets, potentially druggable drivers of cancer [5,6,7,8], including those established on insights into the genetic and molecular heterogeneity of cancer [9]; further refining the dual roles played in cancer by entire families of regulatory biomolecules [10]; and, finally, a conceptually novel approach based on a paradigm shift, with a focus on the characteristic molecular signatures of malignant cells, irrespective of their traditional histopathological classification [11].

Currently, the majority of the global population is exposed to varying levels of environmental carcinogens, such as arsenic, which is present at high levels in drinking water in some regions, asbestos, which was widely used in construction building before being banned in many countries, to name a few. The study by Jasmine et al. investigates the gene–environment interaction in the context of the most common type of skin cancers, namely non-melanoma skin cancers (NMSCs), which comprise basal cell carcinoma (BCC), Bowen’s disease (BD) and invasive squamous cell carcinoma (SCC) [1]. Various environmental factors and exposure to inorganic arsenic in particular are well-established risk factors for NMSC. Jasmine et al. studied a cohort of 32 patients with NMSC, 26 with BCC and 6 with SCC, who were exposed to high or low levels of arsenic from contaminated drinking water. The authors focused on tumor-specific somatic small deletions (DELs), as no study has ever investigated DELs in the context of NMSC and arsenic exposure. A number of tumor-exclusive DELs were identified, including those associated with BCC and SCC, many of them were located in the homopolymeric regions of the genome. For the identified tumor-exclusive DELs, an association was found with transcriptomic changes affecting multiple pathways. Of particular interest is the finding that elevated levels of programmed death ligand 1 (PD-L1), which were previously reported in BCC, are significantly higher in the presence of particular DELs in the subgroup of patients with low arsenic exposure. This makes these patients good candidates for therapy with immune checkpoint inhibitors (ICIs). At the same time, individuals without such DELs who were exposed to high levels of arsenic showed low levels of PD-L1 dysregulation and, consequently, were not good candidates for ICI therapy. The practical take-home messages from this study are: (1) the clinical utility of previously overlooked homopolymeric regions of the genome in the context of NMSC; (2) a proposed strategy to stratify patients for treatment based on their genomic features and their environmental exposure to arsenic. As discussed above, this study unlocks the potential for personalized therapies for BCC/SCC by integrating a patient’s genetic features and environmental exposure to arsenic.

Another well-known environmental carcinogen is asbestos. There is a clear link established between the asbestos exposure and the risk of mesothelioma—a rare and aggressive tumor with a dismal prognosis. Mesothelioma is commonly diagnosed at late stages, and, because of its rarity and the scarcity of relevant animal models, it remains understudied. Therefore, establishing and characterizing such models is of obvious value. Shindyapin et al. describe a xenograft NSG-SGM3 mouse model based on human-derived mesothelioma reporter cell line expressing luciferase and fluorescent protein mCherry [2]. This model was used to study tumor–host interactions, with a focus on myeloid cells. Previously, the prognostic value of the neutrophil-to-lymphocyte ratio in mesothelioma has been established: a high ratio is associated with poor prognosis. In line with this, the post-tumor engraftment analysis in the NSG-SGM3 model demonstrated an increase in neutrophil-like Ly6G+ populations and a decrease in Ly6C+ inflammatory monocytes in the blood of NSG-SGM3 mice with a transplanted tumor, as well as significant infiltration of Ly6C+ cells within the tumor. Chemoattractants for neutrophils were also detected in the tumor tissue. Thus, this model is a suitable tool for studying the mesothelioma microenvironment and tumor–host interactions, with a particular focus on myeloid component.

Moving from the subject of carcinogenic molecules to molecules with anticancer activities, a review by Kashyap et al. comprehensively characterizes the small molecule Metarrestin [3]. This is a first-in-class inhibitor targeting the perinucleolar compartment (PNC). The PNC is a predominantly cancer-specific phenotypic feature contributing to cancer progression and metastasis, with significantly higher presence in metastatic cells. It is enriched with small RNAs transcribed by RNA polymerase III, as well as with RNA-binding proteins that execute pre-mRNA processing. Metarestin was initially identified in a screening of 140,000 compounds for their ability to disassemble the PNC. The first-in-human clinical trial was launched in 2020 to assess its safety and tolerability. Furthermore, Metarestin was shown to bind the translation factors eEF1A1 and eEF1A2, which are involved in a multitude of fundamental processes in the cell. In animal models, it showed excellent permeability and metabolic stability, as well as favorable oral bioavailability. In human plasma, Metarestin demonstrated stability, but toxicity studies indicated some clinical signs of toxicity at mid- and high-dose levels. Moderate cardiac toxicity of Metarestin was computationally estimated. Additionally, other important characteristics of Metarestin that are applicable to its potential clinical use are described in detail by Kashyap et al. in their review. Overall, given that the PNC is a feature of many types of malignancies, the potential applicability of Metarestin is broad, especially as a component of combination therapy. It is thus not surprising that the authors refer to it a “small molecule with big impact” regarding its status as a potential adjuvant treatment for many metastatic cancers.

High metastatic potential, as well as the scarcity of definitive targets for therapy, is a prominent characteristic of triple-negative breast cancer (TNBC). A promising therapeutic approach is to induce cancer cell death by C_6_-ceramide, a sphingolipid with a range of anticancer properties. Previously, a nanopolisome formulation of short-chain C_6_-ceramide (CNLs) was developed, demonstrating biological stability and an absence of toxicity. Furthermore, preclinical tests have shown its therapeutic activity in a variety of solid tumors, including its ability to induce necroptosis in some cancers. In the current study, using a TNBC cell model, Ohya et al. demonstrated pro-necroptotic effect of CNLs in TNBC. They also identified the mixed lineage kinase domain-like protein (MLKL) as a potential TNBC biomarker, based on the observation that its mRNA levels were higher in TNBC tumor tissues and cell lines compared to non-TNBC breast cancer subtypes [4]. Based on the results of gene expression analysis, the authors proposed that the STAT1-IRF1 pathway contributes to elevated levels of MLKL in TNBC cells. Treatment with CNLs induced the relocation of MLKL to cell membrane to form a pore, which is necessary to execute necroptotic cell death, whereas knockdown of MLKL rescued the cells from CNL-induced necroptosis. Hence, this study suggests the clinical utility of MLKL as a TNBC biomarker and proposes CNLs for further evaluation in animal models of TNBC as a pro-necroptotic agent. All of this lays the groundwork for tailored treatments for TNBC.

A work by Ziemiński et al. also explores potential biomarkers in cancer [5], with a focus on endometrial cancer (EC), the most common cancer of the female reproductive system. Using immunohistochemical analysis, Ziemiński et al. compared EC tissues and normal endometrium in a cohort of 60 patients and 60 donors without EC. They found that the level of tumor suppressor TSG101A is much lower in EC tissues compared to non-cancerous endometrium. Therefore, the level of expression of TSG101A might be useful as a diagnostic marker of EC. The authors also demonstrated that low-grade EC is characterized by decreased expression of the transcription factor LSF, which is known to be involved in epithelial–mesenchymal transition (EMT) and cancer cell survival, whereas its increased expression may be a sign of disease progression and a worse prognosis.

While common, EC generally has high survival rate if appropriate treatment is provided. In contrast, breast cancer (BC), the most common cancer among women, is also a leading cause of cancer-related death in this demographic. The study by Aljagthmi investigated the role of decorin (DCN) in BC. DCN is a proteoglycan secreted by breast stromal fibroblasts (BSFs) in the BC’s tumor microenvironment (TME) [6]. The authors found that BC cells are capable of downregulating DCN in BSFs, and that DCN downregulation is observed in active cancer-associated fibroblasts (CAFs). DCN-deficient CAFs promoted EMT, stemness and cisplatin-resistance of BC cells, as well as angiogenesis and orthotopic tumor growth in mice. Specific knockdown of DCN in BSFs stimulated the invasiveness of cancer via STAT3/AUF1 signaling, whereas ectopic expression of DCN somewhat suppressed their pro-carcinogenic functions. Collectively, these data demonstrate that endogenous DCN is a negative regulator of pro-carcinogenic and pro-metastatic effects of BSFs in BC, as well as a mediator of cisplatin resistance—a finding that may inform therapy strategies for BC.

Another mediator of chemoresistance, albeit with an opposing, pro-carcinogenic role, was characterized in work by Poyil et al. [7], who further investigated the role of Polo-like kinase 1 (PLK1) in colorectal cancer (CRC). They confirmed previously reported elevation of PLK1 levels in the majority of CRC cases. Such elevation was associated with an increase in p-ERK1/2, aggressive clinico-pathological parameters, and, as validated in CRC cells, EMT induction, stemness, and resistance to 5-FU treatment. The elevated levels of PLK1 were causative of such resistance, because this resistance was reversed by PLK1 inhibition. Regarding the underlying mechanisms, overexpression of PLK1 in CRC cells led to the activation of CRAF-MEK-ERK signaling, and the inhibition of this signaling mitigated the effects of PLK1. Additionally, the authors found that PLK1 is an independent predictor of lymph node metastasis in CRC. Taken together, these data support a previously proposed idea that PLK1 might be used as a biomarker and a therapeutic target in some subtypes of CRC.

A potential therapeutic target in diverse types of malignant neoplasms, comprehensively reviewed by Yang et al., is tetraspanin 8 (TSPAN8) [8], a member of the tetraspanin family of transmembrane proteins. The primary function of TSPAN8 is to facilitate the formation of specific molecular complexes within the plasma membrane, the so-called “tetraspanin-enriched microdomains (TEMs)” or “tetraspanin nanodomains”. The authors describe the topology and structure of TSPAN8 within these molecular complexes, its post-translational modifications and protein interactome. They report that, intriguingly, TSPAN8 can translocate to the nucleus and act as a transcriptional cofactor promoting the expression of oncogenes. The authors also highlight its significant role in signaling via cancer-derived exosomes and extracellular vesicles, including those originating from cancer stem cells (CSCs). Furthermore, the authors discuss TSPAN8’s involvement in the regulation of several pathways known to be crucial for tumorigenesis, such as Hedgehog, Wnt/β-catenin, EGFR, and mTOR signaling. Overall, TSPAN8 emerges as a “moonlighting” protein with diverse functions, a considerable number of which are linked to tumorigenesis. Given its established role in the immuno-, chemo-, and radiotherapy resistance of tumors, as well as its role in EMT and CSC biology, TSPAN8 is an attractive target in many malignancies and a potential tumor-agnostic target, a concept discussed later in the text.

In the review by Nowacka et al., the focus shifts from individual proteins to the entire protein family of sirtuins (SIRTs) in the context of cancers of the central nervous system (CNS) [9]. SIRTs are seven proteins with class III histone deacetylase activity, as well as deacetylase-independent functions, and versatile biological roles. In cancer, they are involved in chromatin remodeling, CSC biology, the conversion of cells within the TME to a pro-tumor phenotype, cell-cycle regulation, DNA damage repair, mitochondrial and cellular metabolism, oxidative stress management, ribosome biogenesis, apoptosis, and other processes integral to tumorigenesis. Certain functions are common for all SIRTs, but individual SIRTs also have unique roles defined by their structure and subcellular localization (for example, nucleolar localization of SIRT7). Depending on the cellular context and tumor type, they can act as oncogenes or tumor suppressors. In CNS tumors, levels of some SIRTs are significantly elevated or downregulated. The authors summarize that in the context of CNS tumors, SIRT1 and SIRT7 act as oncogenes, SIRT2 and SIRT3 have a rather dual role (oncogenic and tumor-suppressing); SIRT4, SIRT5, and SIRT6 are largely tumor suppressors. Notably, knockdown of SIRT7 not only slows tumor growth in glioma but also enhances the efficacy of temozolomide. Overall, the potential of targeting SIRTs in CNS tumors, summarized by Nowacka et al., warrants further investigation.

While some targets, including TSPAN8 discussed above, appear to be relevant across several cancer types, the clinical, molecular, and genetic heterogeneity of some cancers dictates the need for a precise determination of molecular status, and personalized treatment, with targets specific to a particular subtype. An example of such cancers is synovial sarcoma (SS), a highly aggressive tumor with a 5-year survival rate of <50%. The therapeutic options for SS are very limited and comprise surgery, radiotherapy and standard chemotherapy using cytostatic agents and a small number of receptor tyrosine kinase (RTK) inhibitors. SS is commonly driven by SS18-SSX fusion proteins, resulting from the various t(X;18)(p11.2;q11.2) chromosomal translocations. Alternative fusions (EWSR1-SSX1, MN1-SSX1) and rare mutations in a range of known tumor suppressors/oncogenes may also occur. The aforementioned fusions contribute to carcinogenesis via chromatin remodeling. The course of the disease may vary depending on the type of fusion protein and the genetic landscape. The in-depth review by Lesovaya et al. [10] systematically describes the genetic and epigenetic characteristics of this malignancy, the specific features of its transcriptomic profiles, including the non-coding transcriptome, and key signaling pathways that represent its potential therapeutic targets. Notably, some of these pathways are directly associated with products of the SS18-SSX fusion, and some are not. The authors outline the front-line and second-line therapies for SS, which are currently ongoing main clinical trials testing a range of RTK inhibitors, epigenetic modifiers and immune checkpoint inhibitors, and provide a list of many novel targets for SS therapy, including those based on particular “molecular fingerprints” of the tumor. For example, G3139, an antisense oligodeoxynucleotide against BCL-2, could be an additional therapeutic option for BCL-2-positive synovial sarcomas.

Finally, moving from “cancer heterogeneity” to “cancer uniformity,” a conceptually novel strategy, tumor-agnostic therapy, was discussed by El-Sayed et al. [11]. Indeed, histopathologically diverse and unrelated malignant tumors may share many common genetic and molecular features, the so-called tumor-agnostic markers. For example, it is well known that the gene *TP53*, encoding tumor suppressor p53, is one of the most frequently mutated genes across a variety of cancers. Mutations in genes encoding proteins involved in DNA repair and the maintenance of genome stability, such as *BRCA1/2* and others, are also common in a range of cancers. Further, in many types of unrelated cancers, genes encoding neurotrophic tyrosine kinase receptors (NTRKs) are often found fused with other genes, resulting in aberrant signaling. Other examples of tumor-agnostic markers include the receptor tyrosine kinases ERBB2 (also known as HER2) and RET, as well as proteins involved in signal transduction, such as KRAS and BRAF. Additionally, the TME in many unrelated tumors may share common characteristics, such as features of the extracellular matrix (ECM), the composition of cell populations within the TME, including immune cells, and properties of tumor vasculature. Thus, selecting common targets for therapy in different neoplasms is a reasonable approach, and “basket trials” testing a single agent in patients with different tumors sharing the same “molecular anomaly” gains a value in the context of the tumor-agnostic philosophy. The authors provide a summary of FDA-approved tumor-agnostic therapies, and this list is expected to grow.

## Abbreviations

The following abbreviations are used in this manuscript:

NMSC	Non-melanoma skin cancers
BCC	Basal-cell carcinoma
BD	Bowen’s disease
CSCs	Cancer stem cells
CRC	Colorectal cancer
CAFs	Cancer-associated fibroblasts
DELs	Somatic small deletions
DCN	Decorin
EC	Endometrial cancer
ECM	Extracellular matrix
EMT	Epithelial–mesenchymal transition
MLKL	Mixed lineage kinase domain-like protein
NTRKs	Neurotrophic tyrosine kinase receptors
PD-L1	Programmed death ligand 1
PLK1	Polo-like kinase 1
PNC	Perinucleolar compartment
RTK	Receptor tyrosine kinase
SCC	Squamous-cell carcinoma
SI	Special Issue
SIRTs	Sirtuins
SS	Synovial sarcoma
TEMs	Tetraspanin-enriched microdomains
TNBC	Triple-negative breast cancer
